# Developmental Window-Dependent Effects of Neonatal Tactile Stimulation on Sensorimotor Development in WAG/Rij Rat Pups

**DOI:** 10.3390/life16071152

**Published:** 2026-07-12

**Authors:** Aymen Balikci, Teresa A. May-Benson, Gul Ilbay

**Affiliations:** 1 Sense On Education and Consulting, İstanbul 34810, Türkiye; 2 TMB Educational Enterprises, LLC, Norristown, PA 19401, USA; tmb@tmbeducation.com; 3 Department of Physiology, Faculty of Medicine, Kocaeli University, Kocaeli 41001, Türkiye; gulilbay@yahoo.com

**Keywords:** WAG/Rij rat, neonatal tactile stimulation, sensorimotor development, developmental window, absence epilepsy, neuroplasticity

## Abstract

**Background:** Early-life sensory experiences play a crucial role in the maturation of neural circuits underlying sensorimotor development. The present study investigated whether the effects of neonatal tactile stimulation (NTS) on sensorimotor development depend on the developmental timing of the intervention in WAG/Rij rat pups, a well-established genetic model of absence epilepsy. **Methods:** Seventy rat pups were randomly assigned to five groups: Wistar control, WAG/Rij control, and WAG/Rij rats receiving NTS during the first (PN Week 1-NTS), second (PN Week 2-NTS), or third (PN Week 3-NTS) postnatal week. Tactile stimulation was administered three times daily. Sensorimotor development was assessed on postnatal day 22 using a modified rung-bridge task that evaluated orientation, flexor/extensor activity, distal control, gait development, postural control, sensorimotor responses, tail use, total sensorimotor performance, and crossing time. **Results**: WAG/Rij control pups exhibited impaired sensorimotor performance compared with Wistar controls, demonstrating lower distal control, gait development, sensorimotor response, and total sensorimotor scores, together with prolonged crossing times. The effects of the NTS protocol were dependent on the developmental window in which it was administered. The NTS protocol applied during the second postnatal week was associated with the most pronounced improvements, including significantly higher distal control, sensorimotor response, tail performance, and total sensorimotor scores, as well as significantly shorter crossing times compared with untreated WAG/Rij controls. In contrast, orientation, flexor/extensor activity, and postural control were not significantly affected. **Discussion and Conclusions:** These findings indicate that sensorimotor abnormalities are detectable in WAG/Rij rats during the early postnatal period, before the typical adult expression of absence seizures. The effects of the NTS protocol were strongly dependent on developmental timing, with the second postnatal week representing a particularly sensitive developmental window for sensorimotor maturation. Overall, the NTS protocol was associated with improved early sensorimotor performance in WAG/Rij rats, particularly when administered during the second postnatal week. Because a sham-handled control group was not included, these findings should be interpreted as reflecting the NTS protocol as a whole rather than tactile stimulation alone.

## 1. Introduction

Absence epilepsy is a predominant form of generalized epilepsy characterized by brief episodes of impaired consciousness accompanied by generalized spike-and-wave discharges (SWDs) within thalamocortical networks [1]. Childhood absence epilepsy (CAE) has long been considered a relatively benign epilepsy syndrome because of its favorable seizure prognosis [2]. However, accumulating clinical evidence indicates that many affected children experience cognitive, behavioral, and neuropsychiatric comorbidities rather than just isolated seizures [2,3,4]. Cross-sectional studies have demonstrated high rates of psychiatric disorders, particularly attention-deficit/hyperactivity disorder and anxiety disorders, together with impairments in cognition, language, and social functioning in children with CAE [3]. In addition, children with CAE exhibit significantly higher levels of anxiety and depressive symptoms, as well as psychosocial difficulties such as social isolation and low self-esteem, compared with healthy peers [4]. Collectively, these clinical findings suggest that CAE is associated with more widespread disturbances of brain development and function than the generation of seizures alone [2]. Consistent with these clinical observations in children, experimental studies in WAG/Rij rats have demonstrated comparable cognitive, behavioral, and affective abnormalities, supporting the translational relevance of this animal model for investigating the mechanisms underlying absence epilepsy and its associated comorbidities [1,5,6].

The Rijswijk (WAG/Rij) strain of Wistar Albino Glaxo rats is one of the best genetically characterized models of absence epilepsy. Rats develop spontaneous SWDs in an age-dependent manner and show several behavioral and cognitive abnormalities related to epileptogenesis, similar to patients with childhood absence epilepsy [6,7]. Previous longitudinal EEG studies have demonstrated that the age-related increase in the propensity of WAG/Rij rats to have seizures is consistent with the hypothesis that absence epilepsy evolves over a long maturation process rather than emerging suddenly [7]. Additionally, the expression of seizures and related neuropsychiatric outcomes in this strain is influenced by early developmental environmental factors [1,8].

Early postnatal life in rats is a critical period during which sensory experience shapes the establishment of neuronal connectivity, synaptic organization, and behavioral development. Among the sensory modalities, tactile stimulation is particularly important, as maternal licking, grooming, and physical contact provide key signals for normal neurodevelopment [5,9]. Long-lasting effects on emotional behavior, stress responsivity, learning, memory, and neural plasticity have been demonstrated by experimental studies using early tactile enrichment, neonatal handling, and environmental stimulation [10,11,12]. These beneficial effects are believed to be mediated by changes in neurotrophic signaling, synaptic remodeling, regulation of the hypothalamic–pituitary–adrenal axis, and activity-dependent neuroplasticity [11].

In rodents, studies indicate that early sensory experiences affect not only cognitive development but also the maturation of sensorimotor systems. Motor development in rats is highly structured with sequential gains in posture, gait, limb coordination, distal motor control, and sensorimotor integration in the first weeks after birth [13,14]. Experimental manipulations of the sensory environment can have profound effects on the timing and quality of these developmental processes. Sensory-enriched environments foster the emergence of complex locomotor behaviors, while sensory deprivation may delay or modify the normal developmental trajectories [14,15]. Likewise, sensory feedback has been shown to play an active role in spontaneous motor activity even at the earliest postnatal stages, implying that sensorimotor development relies on ongoing interactions between sensory input and motor output [16].

Studies of early-life experience in rodents also provide evidence of developmental plasticity in motor systems. Increased postnatal sensory exposure improves bilateral coordination, righting responses, and sensory-evoked motor behaviors in neonatal rats [15,17], demonstrating how minor alterations in early experience can affect motor development [18]. Developmental analyses have also shown that some postnatal periods are characterized by rapid changes in gait patterns, postural control, and skilled motor performance, suggesting sensitive developmental periods during which neural circuits are particularly susceptible to environmental inputs [13].

Tactile stimulation in the neonatal period has been suggested as a disease-modifying intervention, in addition to its effects on normal development, in rodent models of genetic absence epilepsy. Previous studies demonstrated that neonatal tactile stimulation decreased the incidence of SWD and improved depression-like behaviors in adult WAG/Rij rats [5]. Furthermore, neonatal tactile stimulation induces long-lasting structural changes in the somatosensory cortex, including decreased dendritic spine density in layer V pyramidal neurons, a cortical area critically involved in the generation of absence seizures [11]. These results suggest that tactile stimulation may affect epileptogenesis by modulating cortical maturation and neural plasticity.

The influence of neonatal tactile stimulation on the development of early sensorimotor skills in WAG/Rij offspring remains poorly understood, although a growing body of evidence indicates its beneficial effects on seizure susceptibility, emotional behavior, and cortical morphology. This question is particularly relevant because sensorimotor development occurs during the same developmental window in which tactile stimulation has its most powerful neuroplastic effects. Moreover, previous studies have suggested that environmental interventions may have differential effects depending on the developmental stage in which they are applied, indicating the importance of developmental timing [1,19].

Thus, the aim of the present study was to investigate the effects of neonatal tactile stimulation on sensorimotor development in WAG/Rij rat pups across developmental windows. We hypothesized that tactile stimulation during selective postnatal developmental windows would improve sensorimotor performance and accelerate the maturation of motor behaviors, reflecting an increased neuroplastic adaptation during early postnatal development.

## 2. Materials and Methods

### 2.1. Animals and Experimental Design

All experimental procedures were approved by the Kocaeli University Animal Experiments Local Ethics Committee (KOU HADYEK, Approval No. 10/4-2025) and were conducted in accordance with national and international guidelines for the care and use of laboratory animals. Ethical approval was granted on 27 October 2025.

WAG/Rij rats and non-epileptic Wistar rats were obtained from the breeding colony of the Kocaeli University Experimental Medicine Research and Application Center (DETAB, Kocaeli, Türkiye). Animals were maintained under standard laboratory conditions (12 h light/dark cycle; lights on at 07:00 h; temperature 21 ± 2 °C) with ad libitum access to food and water.

Seventy rat pups were assigned to one of five experimental groups (*n* = 14 per group): Wistar control, WAG/Rij control, WAG/Rij neonatal tactile stimulation (NTS) during the first postnatal week (PN Week 1-NTS), WAG/Rij neonatal tactile stimulation during the second postnatal week (PN Week 2-NTS), and WAG/Rij neonatal tactile stimulation during the third postnatal week (PN Week 3-NTS). Randomization was performed using a two-step procedure. First, dams (litters) were randomly assigned to the experimental groups. Each experimental group consisted of offspring originating from three independent dams (three separate litters), ensuring that no group was derived from a single litter and thereby reducing the potential influence of litter-specific maternal and environmental effects. Following litter allocation, pups were selected from the assigned litters to establish comparable group sizes. Group size was predetermined to be 14 pups per group based on offspring availability and the potential for attrition during the preweaning period. During allocation, efforts were made to maintain a balanced sex distribution across groups. Based on the available offspring within the selected litters, each group ultimately consisted of 8 male and 6 female pups. Thus, allocation was balanced with respect to both litter origin and sex distribution, with all groups containing offspring from three independent litters and an identical male-to-female ratio. Natural litter sizes in the breeding colony were 6–8 pups per dam. Litter size was not standardized by culling, as reducing litter size may alter mother care, nursing behaviors, and early postnatal development, thus introducing another experimental variable. During group allocation, dams were selected with litter sizes as close as possible to one another within the experimental groups to avoid potential confounding by litter size. Throughout the preweaning period, pups remained with their dams in their home cages to minimize potential stress associated with cage transfer and environmental novelty. Sensorimotor assessment was performed on postnatal day 22 (PND22).

Both male and female pups were included in all experimental groups. The primary objective of the study was to investigate developmental window-dependent effects of neonatal tactile stimulation on sensorimotor development rather than sex-specific differences. Therefore, sex was not included as an independent factor in the statistical analyses. In addition, the sample size was determined to be inadequate to provide sufficient statistical power for sex-stratified analyses. Consequently, data from male and female pups were pooled for the primary analyses. This approach is consistent with previous developmental studies with comparable sample sizes, in which sex was controlled through balanced allocation but not analyzed as a separate experimental factor [13].

### 2.2. Neonatal Tactile Stimulation

**Figure 1:**
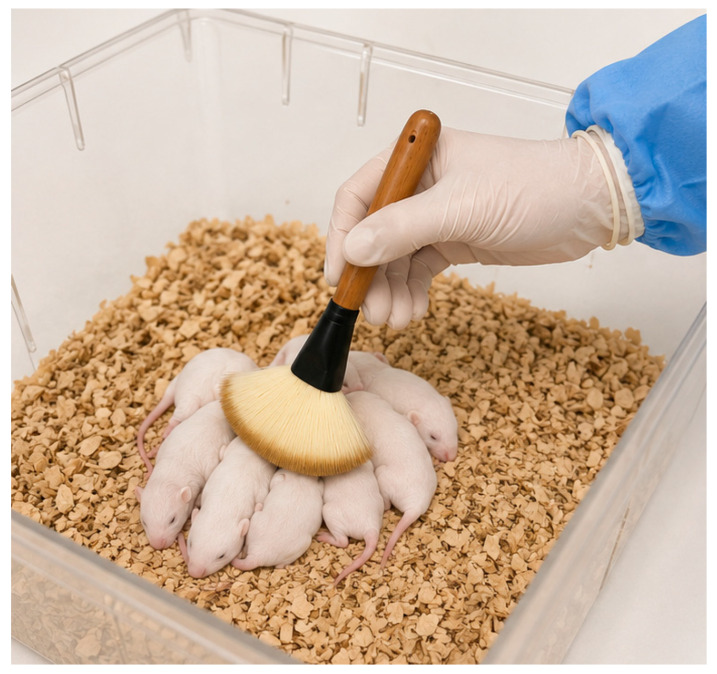
Representative illustration of the neonatal tactile stimulation (NTS) procedure and the soft brush used in the study.

Neonatal tactile stimulation (NTS) was administered during different developmental windows to investigate potential developmental window-dependent effects on sensorimotor maturation. Pups were gently removed from the home cage and placed in a temperature-controlled environment. Tactile stimulation was administered with a soft-bristled brush with a cylindrical handle (approximately 2 cm in diameter) and a broad, flat brush tip roughly 8 cm wide. The brush was made of fine, soft bristles (about 4–5 cm in length) to provide gentle, consistent tactile stimulation during the session (Figure 1).

Stimulation sessions lasted 15 min and were administered three times daily (09:00, 12:00, and 15:00) by the same experimenter to ensure procedural consistency. Neonatal tactile stimulation was delivered during distinct postnatal developmental windows corresponding to the first, second, or third postnatal week. Control animals remained with their dams and were not exposed to any experimental manipulation other than routine husbandry procedures. The stimulation protocol was adapted from previously published neonatal tactile stimulation paradigms [5].

### 2.3. Sensorimotor Assessment

Sensorimotor development was assessed using a modified rung-bridge stepping task based on the skilled walking paradigm of Metz and Whishaw and the developmental scoring system described by Shriner et al. (2009) [13]. To implement this assessment, we designed and constructed a custom-built rung-bridge apparatus in our laboratory using the apparatus reported by Shriner et al. (2009) as the structural basis, with modifications for the present study [13]. The apparatus measured 100 cm in length, 20 cm in height, and 8 cm in width and consisted of 77 metal rungs (3 mm in diameter). The apparatus was elevated 1 cm above the supporting surface. The irregular rung pattern was based on a repeating 49 cm sequence comprising an initial 20 cm section with 1 cm inter-rung gaps, followed by five 2 cm gaps, ten 1 cm gaps, and three 3 cm gaps. This sequence was repeated twice along the 100 cm runway, leaving approximately 2 cm of terminal space at the ends of the apparatus. The same fixed rung configuration was used for all animals throughout the study.

Each animal was placed individually on the apparatus and allowed to traverse the rung bridge while being video-recorded. Behavioral recordings were subsequently analyzed frame-by-frame by an observer blinded to the experimental groups. In addition to sensorimotor scoring, the time required to cross the entire apparatus (crossing time) was recorded for each animal. A maximum trial duration of 180 s was allowed; animals that failed to complete the task within this period were assigned a crossing time of 180 s [13,15].

The primary outcomes of the study were the total sensorimotor score and crossing time, which were selected as global indicators of sensorimotor performance and maturation. Individual domain scores obtained from the modified rung-bridge assessment (orientation, flexor/extensor activity, distal control, gait development, postural control, sensorimotor responses, and tail performance) were analyzed to characterize specific components of sensorimotor development and were considered exploratory component analyses of the overall assessment.

### 2.4. Behavioral Scoring for Modified Rung-Bridge Stepping Task

Behavioral performance was evaluated using a modified, developmental, skilled walking rating system adapted from Shriner et al. (2009) [13]. Video-recordings were analyzed frame-by-frame, and sensorimotor function was assessed across seven behavioral domains: orientation, flexor/extensor activity, gait development, postural control, sensorimotor responses, distal limb control, and tail use. Each behavioral variable was scored using a three-point ordinal scale, where 0 indicated absent or immature performance, 1 indicated partially developed or inconsistent performance, and 2 indicated mature, adult-like performance. Scores were normalized to the number of analyzed steps, averaged for each animal, and summed to obtain both domain-specific scores and an overall sensorimotor performance score.

### 2.5. Orientation Score

Orientation included evaluation of head, trunk, and limb orientation. Head orientation assessed whether the head was directed toward the direction of movement progression. Trunk orientation evaluated the alignment of the body axis with the direction of locomotion. Limb orientation assessed the limbs’ position relative to the trunk during stepping. For each parameter, a score of 0 indicated marked deviation from the expected orientation, 1 indicated partial alignment, and 2 indicated consistent alignment with the direction of movement.

### 2.6. Flexor/Extensor Activity Score

Flexor/extensor activity included assessment of paw flexion, paw extension, and digit closure during limb lifting. Paw flexion evaluated the flexion of the wrist during limb elevation. Paw extension assessed the extension of the paw and digits before contacting the rung. Digit closure evaluated the coordinated closing of the digits as the limb was lifted from the supporting surface. A score of 0 indicated absent or poorly organized movement, 1 indicated incomplete execution of the movement, and 2 indicated complete and coordinated execution.

### 2.7. Gait Development Score

Gait development included evaluation of forelimb and hindlimb placement, forelimb and hindlimb abduction, forelimb and hindlimb adduction, and coordinated stepping. Forelimb and hindlimb placement assessed the animal’s ability to accurately position each paw onto the intended rung. Abduction evaluated excessive lateral movement of the limb away from the body during stepping, whereas adduction assessed inappropriate movement toward the body midline. Coordinated stepping evaluated temporal coordination between forelimb and hindlimb movements during forward locomotion. A score of 0 indicated absent or unsuccessful placement with poor coordination, 1 indicated partially coordinated stepping with occasional successful placement, and 2 indicated accurate rung placement together with coordinated forelimb–hindlimb locomotion.

### 2.8. Postural Control Score

Postural control included assessment of weight bearing, base of support, and limb placement on the rung. Weight bearing evaluated the animal’s ability to elevate and support the trunk during locomotion. Base of support assessed hindlimb positioning relative to the body axis as an indicator of postural stability. Limb placement on the rung was used to evaluate the orientation and stability of the paw after contacting the rung. A score of 0 indicated inability to adequately support body weight or maintain stable posture, 1 indicated partial postural control, and 2 indicated stable trunk support together with appropriate limb positioning throughout locomotion.

### 2.9. Sensorimotor Response Score

Sensorimotor responses included assessment of placing response, footfall recovery, foot-slip recovery, stepping between rungs, adjustment on the rung, and aiming for the target rung. Placing response evaluated the ability to accurately position the paw onto a rung during forward progression. Footfall recovery assessed the animal’s ability to regain support after the paw slipped completely between two rungs. Foot-slip recovery evaluated rapid correction following partial loss of paw contact with a rung. Stepping between rungs assessed whether the animal appropriately generated a corrective step after landing in the space between adjacent rungs. Adjustment on the rung evaluated repositioning of the paw after initial contact to obtain a more secure foothold. Aim for the rung assessed the accuracy with which the paw was directed toward the intended rung before contact. For all sensorimotor response variables, a score of 0 indicated absent or ineffective corrective behavior, 1 indicated delayed or incomplete correction, and 2 indicated rapid, accurate, and successful execution of the corrective response.

### 2.10. Distal Control Score

Distal limb control included evaluation of digit use, grip flexion, and arpeggio movement. Digit use assessed the number of digits effectively engaged during grasping of the rung. Grip flexion evaluated the ability of the digits and paw to securely grasp the rung after contact. Arpeggio movement assessed sequential engagement of digits 5 through 2 around the rung together with wrist pronation during paw placement, reflecting mature distal motor control. A score of 0 indicated absent grasping or flat paw placement, 1 indicated partial digit recruitment with incomplete grip formation, and 2 indicated complete digit engagement together with firm grip flexion and mature arpeggio movement.

### 2.11. Tail Use Score

Tail use evaluated tail position during locomotion. Tail posture was assessed according to its contribution to balance while traversing the apparatus. A score of 0 indicated that the tail remained low and inactive, 1 indicated intermittent elevation during stepping, and 2 indicated consistent active positioning of the tail, contributing to postural stability throughout locomotion.

### 2.12. Statistical Analysis

Statistical analyses were performed using GraphPad Prism version 10.5 (GraphPad Software, San Diego, CA, USA).Group differences were evaluated using the nonparametric Kruskal–Wallis test followed by Dunn’s multiple-comparison post hoc test. Exact adjusted p-values are reported for relevant pairwise comparisons. Effect sizes for overall group differences were estimated using epsilon-squared (ε^2^). Data are presented as mean ± standard error of the mean (SEM) in the figures, whereas median and interquartile range (Q1–Q3) values are provided in the Supplementary Material. Statistical significance was defined as *p* < 0.05.

## 3. Results

### 3.1. Primary Outcomes

#### 3.1.1. Total Sensorimotor Score

**Figure 2:**
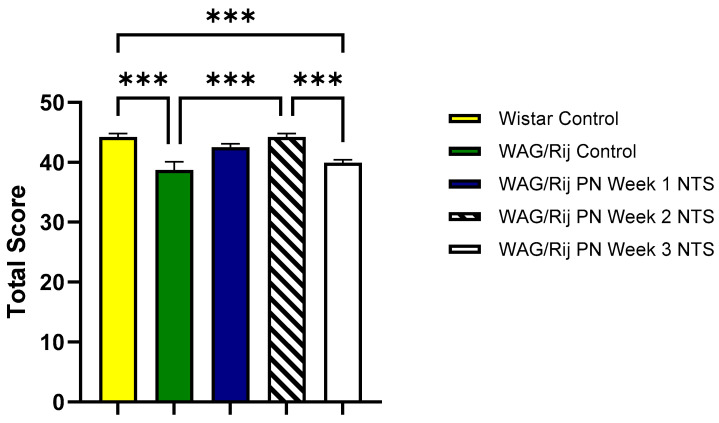
Total sensorimotor scores of Wistar Control, WAG/Rij Control, PN Week 1-NTS, PN Week 2-NTS, and PN Week 3-NTS groups. Total score was calculated as the sum of orientation, flexor/extensor activity, distal control, gait development, postural control, sensorimotor response, and tail scores. Data are presented as mean ± SEM. Significant pairwise comparisons (Dunn’s post hoc test): Wistar Control vs. WAG/Rij Control (*** *p* < 0.001), WAG/Rij Control vs. PN Week 2-NTS (*** *p* < 0.001), and PN Week 2-NTS vs. PN Week 3-NTS (***p < 0.001), WAG/Rij Control vs. PN Week 3-NTS (*** *p* < 0.001).

A significant overall group effect was observed for total sensorimotor score (Kruskal–Wallis test: H = 31.82, *p* < 0.0001, ε^2^ = 0.435; see Supplementary Material). Post hoc Dunn’s multiple-comparison analysis revealed that WAG/Rij control pups displayed significantly lower total sensorimotor scores than Wistar controls (adjusted *p* = 0.0009). Neonatal tactile stimulation administered during the second postnatal week significantly increased total sensorimotor performance compared with WAG/Rij controls (adjusted *p* = 0.0009) and PN Week 3-NTS animals (adjusted *p* = 0.0005). Total sensorimotor scores in the PN Week 3-NTS group remained significantly lower than those of Wistar controls (adjusted *p* = 0.0005) (Figure 2).

#### 3.1.2. Crossing Time

**Figure 3:**
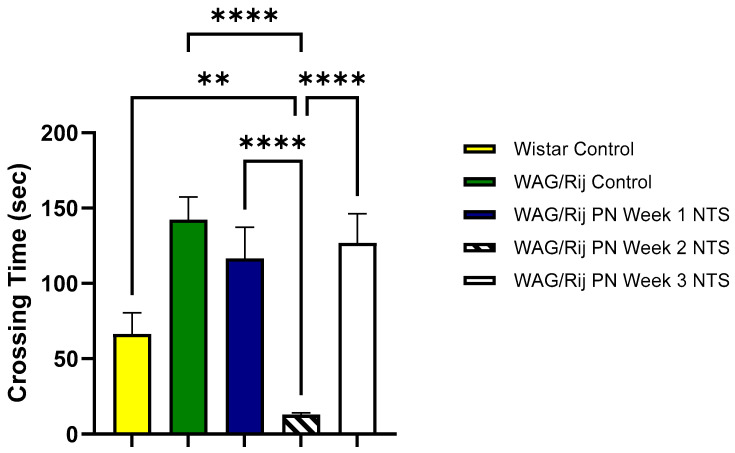
Crossing time required to traverse the rung bridge apparatus in Wistar Control, WAG/Rij Control, PN Week 1-NTS, PN Week 2-NTS, and PN Week 3-NTS groups. Data are presented as mean ± SEM. Significant pairwise comparisons (Dunn’s post hoc test): PN Week 2-NTS vs. Wistar Control (** *p* < 0.01), PN Week 2-NTS vs. WAG/Rij Control (**** *p* < 0.0001), PN Week 2-NTS vs. PN Week 1-NTS (**** *p* < 0.0001), and PN Week 2-NTS vs. PN Week 3-NTS (**** *p* < 0.0001).

Crossing time differed significantly among groups (Kruskal–Wallis test: H = 38.71, *p* < 0.0001, ε^2^ = 0.542; see Supplementary Material). Post hoc Dunn’s multiple-comparison analysis revealed that the PN Week 2-NTS group required significantly less time to traverse the apparatus than Wistar controls (adjusted *p* = 0.0064), WAG/Rij controls (adjusted *p* = 0.0001), PN Week 1-NTS animals (adjusted *p* = 0.0001), and PN Week 3-NTS animals (adjusted *p* = 0.0001). No significant differences were detected between WAG/Rij controls and either the PN Week 1-NTS or PN Week 3-NTS groups (Figure 3).

### 3.2. Domain-Specific Outcomes

#### 3.2.1. Orientation, Flexor/Extensor Activity, and Postural Control

**Figure 4:**
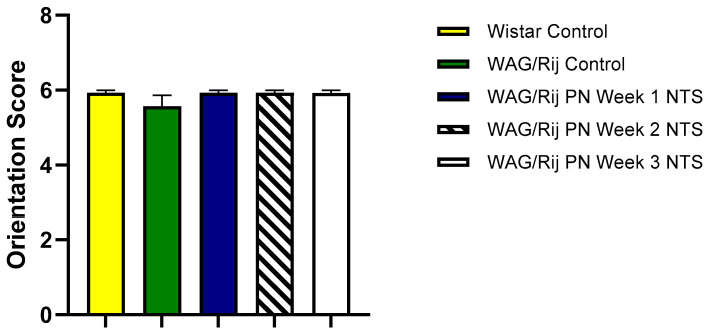
Orientation scores of Wistar control, WAG/Rij control, and neonatal tactile stimulation groups. No significant differences were detected among the groups (Dunn’s post hoc test, all *p* > 0.05).

**Figure 5:**
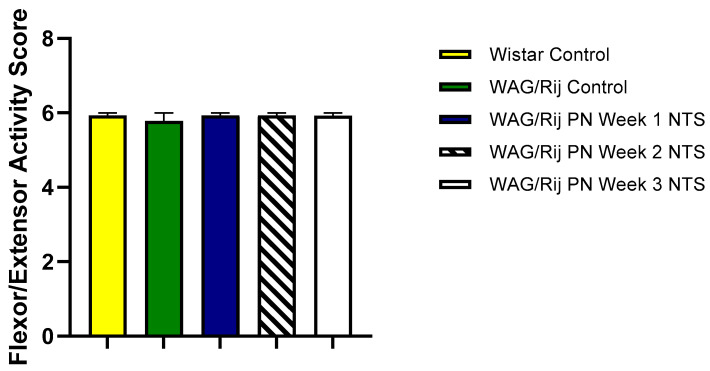
Flexor/extensor activity scores of Wistar control, WAG/Rij control, and neonatal tactile stimulation groups. No significant differences were detected among the groups (Dunn’s post hoc test, all *p* > 0.05).

**Figure 6:**
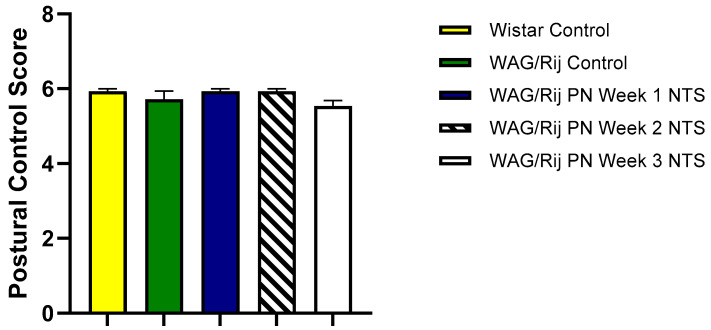
Postural control scores of Wistar control, WAG/Rij control, and neonatal tactile stimulation groups. No significant differences were detected among the groups (Dunn’s post hoc test, all *p* > 0.05).

No significant group differences were observed in orientation score (Kruskal–Wallis test: H = 0.90, *p* = 0.9246, ε^2^ = 0.000), flexor/extensor activity score (H = 0.007, *p* > 0.9999, ε^2^ = 0.000), or postural control score (H = 0.273, *p* = 0.9914, ε^2^ = 0.000; see Supplementary Material). Although minor variations in group values were observed, neonatal tactile stimulation administered during different developmental windows did not significantly affect these parameters compared with either the Wistar or WAG/Rij control groups (Figure 4, Figure 5 and Figure 6).

#### 3.2.2. Distal Control

**Figure 7:**
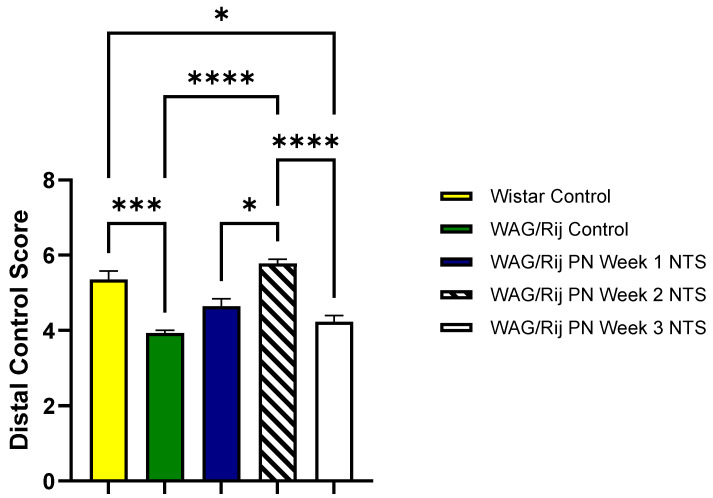
Distal control scores of Wistar Control, WAG/Rij Control, PN Week 1-NTS, PN Week 2-NTS, and PN Week 3-NTS groups. Data are presented as mean ± SEM. Significant pairwise comparisons (Dunn’s post hoc test): Wistar Control vs. WAG/Rij Control (*** *p* < 0.001), WAG/Rij Control vs. PN Week 2-NTS (**** *p* < 0.0001), PN Week 1-NTS vs. PN Week 2-NTS (* *p* < 0.05), and PN Week 2-NTS vs. PN Week 3-NTS (**** *p* < 0.0001), Wistar Control vs. PN Week 1-NTS (* *p* < 0.05).

A significant group effect was observed for distal control scores (Kruskal–Wallis test: H = 10.77, *p* = 0.0293, ε^2^ = 0.106; see Supplementary Material). Post hoc Dunn’s multiple-comparison analysis revealed that WAG/Rij control pups exhibited significantly lower distal control scores than Wistar controls (adjusted *p* = 0.0003). Neonatal tactile stimulation administered during the second postnatal week significantly improved distal control scores compared with WAG/Rij controls (adjusted *p* < 0.0001) and PN Week 3-NTS animals (adjusted *p* < 0.0001). In addition, distal control scores were significantly higher in the PN Week 2-NTS group than in the PN Week 1-NTS group (adjusted *p* = 0.0127) (Figure 7).

#### 3.2.3. Gait Development

**Figure 8:**
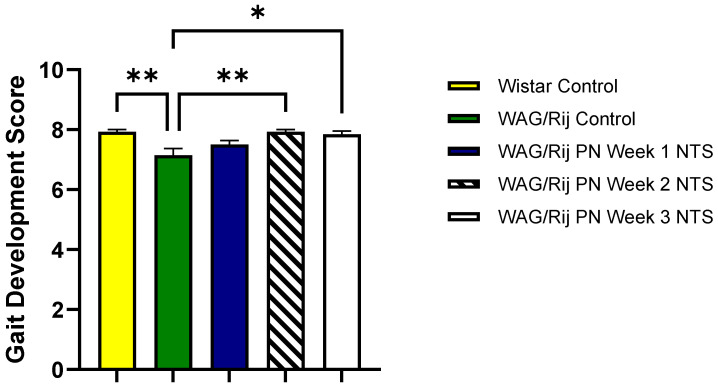
Gait development scores of Wistar Control, WAG/Rij Control, PN Week 1-NTS, PN Week 2-NTS, and PN Week 3-NTS groups. Data are presented as mean ± SEM. Significant pairwise comparisons (Dunn’s post hoc test): Wistar Control vs. WAG/Rij Control (** *p* < 0.01), WAG/Rij Control vs. PN Week 2-NTS (** *p* < 0.01), and WAG/Rij Control vs. PN Week 3-NTS (* *p* < 0.05).

A significant group effect was observed for gait development scores (Kruskal–Wallis test: H = 19.57, *p* = 0.0006, ε^2^ = 0.243; see Supplementary Material). Post hoc Dunn’s multiple-comparison analysis revealed that WAG/Rij control pups exhibited significantly lower gait development scores than Wistar controls (adjusted *p* = 0.0060). Neonatal tactile stimulation administered during the second postnatal week significantly improved gait development scores compared with WAG/Rij controls (adjusted *p* = 0.0060). Similarly, gait development scores were significantly higher in the PN Week 3-NTS group than in WAG/Rij controls (adjusted *p* = 0.0353). No significant differences were detected between the PN Week 1-NTS group and WAG/Rij controls (Figure 8).

#### 3.2.4. Sensorimotor Responses

**Figure 9:**
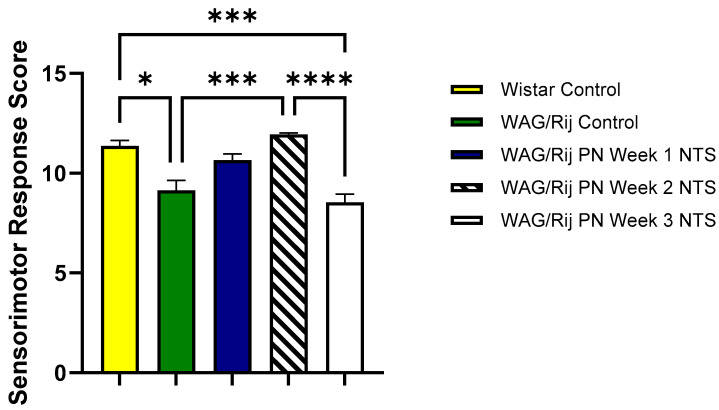
Sensorimotor response scores of Wistar Control, WAG/Rij Control, PN Week 1-NTS, PN Week 2-NTS, and PN Week 3-NTS groups. Data are presented as mean ± SEM. Significant pairwise comparisons (Dunn’s post hoc test): Wistar Control vs. WAG/Rij Control (* *p* < 0.05), WAG/Rij Control vs. PN Week 2-NTS (*** *p* < 0.001), PN Week 2-NTS vs. PN Week 3-NTS (**** *p* < 0.0001), and Wistar Control vs. PN Week 3-NTS (*** *p* < 0.001).

A significant group effect was observed for sensorimotor response scores (Kruskal–Wallis test: H = 37.23, *p* < 0.0001, ε^2^ = 0.519; see Supplementary Material). Post hoc Dunn’s multiple-comparison analysis revealed that WAG/Rij control pups exhibited significantly lower sensorimotor response scores than Wistar controls (adjusted *p* = 0.0102). Neonatal tactile stimulation administered during the second postnatal week significantly improved sensorimotor response scores compared with WAG/Rij controls (adjusted *p* = 0.0001). In addition, sensorimotor response scores were significantly higher in the PN Week 2-NTS group than in the PN Week 3-NTS group (adjusted *p* < 0.0001). Sensorimotor response scores in the PN Week 3-NTS group remained significantly lower than those of Wistar controls (adjusted *p* = 0.0005) (Figure 9).

#### 3.2.5. Tail Score

**Figure 10:**
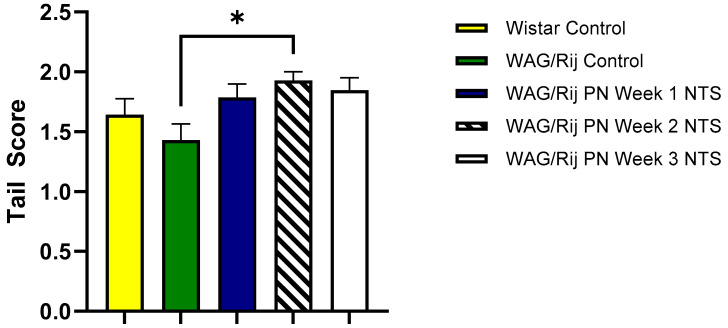
Tail scores of Wistar Control, WAG/Rij Control, PN Week 1-NTS, PN Week 2-NTS, and PN Week 3-NTS groups. Data are presented as mean ± SEM. Significant pairwise comparison (Dunn’s post hoc test): WAG/Rij Control vs. PN Week 2-NTS (* *p* < 0.05).

A significant group effect was observed for tail scores (Kruskal–Wallis test: H = 10.61, *p* = 0.0314, ε^2^ = 0.103; see Supplementary Material). Post hoc Dunn’s multiple-comparison analysis revealed that the PN Week 2-NTS group exhibited significantly higher tail scores than WAG/Rij controls (adjusted *p* = 0.0238). No other pairwise comparisons reached statistical significance (Figure 10).

## 4. Discussion

In the present study, we examined whether the effect of the NTS protocol on sensorimotor development depends on the timing of the intervention during postnatal development in WAG/Rij rat pups. Results found that sensorimotor performance was impaired in WAG/Rij control pups on postnatal day 22 compared with age-matched Wistar controls. Specifically, WAG/Rij pups had lower scores of distal control, gait development, sensorimotor response, and total sensorimotor scores and increased crossing times, reflecting delayed maturation of several sensorimotor functions. The present findings indicate that in this genetic model of absence epilepsy, sensorimotor abnormalities are already present at the early postnatal stage. In addition, the effects of the NTS protocol varied across developmental windows. The best improvements in distal motor control, sensorimotor responses, overall sensorimotor performance, and crossing time were observed during tactile stimulation in the second postnatal week of the stimulation periods studied. Stimulation in the first or third postnatal weeks had limited or inconsistent effects. The findings support the hypothesis that specific developmental windows are particularly sensitive to sensory experiences and suggest that the second postnatal week is a critical period where tactile stimulation is most effective in promoting sensorimotor maturation in WAG/Rij rats.

The first important finding of the present study was the poorer sensorimotor performance of WAG/Rij control pups compared with age-matched Wistar controls at PND22, particularly in distal control, gait development, sensorimotor responses, total sensorimotor score, and crossing time. To our knowledge, this is the first study to demonstrate early postnatal sensorimotor differences between WAG/Rij and Wistar rat pups prior to the typical adult expression of absence seizures. This observation may suggest that the WAG/Rij phenotype is not only confined to the later-life expression of seizures but may also encompass early developmental changes in sensorimotor maturation. This interpretation is consistent with previous evidence that WAG/Rij rats are a genetic model of absence epilepsy, as well as a model associated with behavioral and cognitive comorbidities [6]. WAG/Rij rats are also characterized by reduced sensorimotor gating compared with Wistar controls, suggesting abnormal sensorimotor gating in this strain [20]. Our present findings add to these observations by showing that sensorimotor changes can be detected already in early postnatal development.

Somatosensory–motor and thalamocortical networks are involved in the WAG/Rij phenotype, which may account for these early sensorimotor differences. Absence seizures have been strongly linked to dysfunction of the cortico–thalamo–cortical circuit, and the somatosensory cortex has been identified as an important cortical area for the initiation and propagation of the spike-wave discharges in the genetic models of absence epilepsy [21,22]. Similar findings were reported in previous studies in WAG/Rij rats, where structural changes were observed in layer V pyramidal neurons of the somatosensory cortex, with adult WAG/Rij rats displaying higher dendritic spine densities than non-epileptic Wistar controls [11]. Given the importance of the somatosensory cortex in tactile processing, limb placement, and sensorimotor integration, early deficits within this cortical network may underlie the reduced distal control, poorer sensorimotor responses, and longer crossing times observed in WAG/Rij pups in the present study. Further evidence for this view comes from studies showing that the rodent somatosensory cortex integrates sensory and motor signals necessary for active tactile exploration and goal-directed behavior [23] and that developing somatosensory and motor cortices undergo age-dependent changes in sensory processing and functional connectivity [24].

The fact that WAG/Rij pups display sensorimotor impairment at PND22 is particularly significant given that mature spike-wave discharges in WAG/Rij rats typically manifest later, around 2–3 months of age, and are fully developed in adulthood [8,11]. Therefore, the current findings suggest that sensorimotor impairments may precede overt manifestations of epileptic seizures and may represent early developmental features of the genetic absence epilepsy phenotype. This interpretation is also consistent with reports on functional network alterations in genetic absence epilepsy models at early ages, supporting the idea that abnormal network organization might be present prior to the chronic expression of seizures [25]. These results suggest that early sensorimotor testing may detect subtle developmental abnormalities in WAG/Rij rats and may provide a behavioral window into the development of cortico–thalamo–cortical and somatosensory–motor circuits involved in absence epilepsy.

Another interesting finding was that there were no significant group differences in orientation, flexor/extensor activity, and postural control scores. One explanation may be when the assessment was administered developmentally. In this study, sensorimotor performance was assessed on PND22 when rats had already reached a relatively mature level of several basic motor and postural functions. Previous developmental studies have demonstrated that most basic sensorimotor capacities develop during the second and third postnatal weeks, with more complex motor skills developing thereafter. For example, Shriner et al. (2009) showed that the largest change in mature locomotor behavior occurs between P15 and P19, with many fundamental elements of skilled walking showing considerable development before PND22 [13]. Similarly, developmental studies examining locomotion and posture have demonstrated that the fundamental postural and locomotor behaviors arise early in the first two postnatal weeks and become more stable as pups reach weaning age [14]. Moreover, developmental milestone studies suggest that several sensorimotor functions are usually acquired between P10 and P20, indicating that by PND22 most healthy pups have already mastered these rudimentary developmental skills [26].

This interpretation is also consistent with broader evidence that sensorimotor systems mature along distinct developmental trajectories, with basic sensory and motor functions becoming largely functional by the end of the third postnatal week [27]. Similarly, Jamon (2006) suggested that basic locomotion requires basic neural and musculoskeletal components that are relatively early in development, while later maturation mainly involves refinement of more complex motor behaviors [28]. Therefore, the absence of large differences in orientation, flexor/extensor activity, and postural control may reflect a ceiling effect, given that testing occurred at a more advanced developmental level. These relatively early-maturing functions may have already reached near-maximal performance levels in most animals by PND22, making the assessment less sensitive to detecting strain- or treatment-related differences. However, more complex aspects of sensorimotor performance, such as distal control, sensorimotor responses, gait refinement, and overall task performance, may still be sufficiently dynamic at this age to demonstrate the developmental abnormalities associated with the WAG/Rij phenotype, as well as the benefits of neonatal tactile stimulation.

The beneficial effects of the NTS protocol observed in the present study appeared to be more evident in specific sensorimotor domains than in others. The NTS protocol resulted in significant improvements in distal control, gait development, sensorimotor responses, tail use, and overall sensorimotor performance, whereas orientation, flexor/extensor activity, and postural control were not significantly affected. This pattern is broadly consistent with previous developmental studies indicating that different components of sensorimotor function follow distinct maturational trajectories. Shriner et al. (2009) reported that skilled locomotion develops in sequential stages, with major transitions in gait maturation occurring between P15 and P19 and further refinement continuing beyond this period [13]. Accordingly, the domains that showed improvement following the NTS protocol may represent aspects of sensorimotor performance that remain responsive to environmental influences during the developmental period examined [13,14,15].

The improvement in distal control is especially meaningful, as this domain requires fine regulation of paw and digit movements during contact with the rung. Such performance relies on tactile feedback, proprioceptive information, grip adjustment, and the precise integration of sensory information with motor output [16,29]. Early motor behavior is not a maturational process per se but is strongly influenced by sensory experience [15,30]. Brumley et al. (2015) showed that coordinated action patterns in perinatal rats exhibit considerable developmental plasticity and are sensitive to sensory feedback [29]. Brumley and Robinson (2013) similarly showed that proprioceptive feedback can modulate spontaneous limb movements even in newborn rats [16]. Thus, the increased distal control scores observed after NTS may indicate improved sensory-guided limb placement and more effective tactile-proprioceptive integration.

The positive effect of the NTS protocol on gait development may also reflect experience-dependent facilitation of the maturation of locomotor circuits. Sensory-enriched environments have been shown to influence postural and locomotor expression in juvenile rats [14], suggesting that sensory context contributes to the emergence of motor patterns. These studies, taken together, support the hypothesis that repetitive tactile input in early life may enhance the maturation of coordinated stepping, limb placement, and gait organization.

The increase in sensorimotor response scores is also consistent with the recognized importance of touch during early neurodevelopment. Touch is one of the earliest and most biologically relevant sensory inputs in rodents and is normally provided through maternal licking, grooming, nursing contact, and interactions with littermates. Ardiel and Rankin (2010) highlighted the role of mechanosensory stimulation in promoting growth, behavioral maturation, and neural development across species [9]. Fernández-Teruel et al. (2022) similarly suggested that early enriched sensory experiences, including tactile stimulation and neonatal handling, may produce long-lasting neurobehavioral effects in rodents and preterm infants [10]. Within this context, the improved sensorimotor response scores observed following the NTS protocol are consistent with the possibility that early tactile experience influences aspects of sensorimotor development.

The timing of stimulation during development appeared to be an important factor in the observed outcomes. The most pronounced improvements were observed following stimulation during the second postnatal week, indicating that this developmental period may be particularly responsive to tactile stimulation with respect to the sensorimotor measures examined in the present study. Previous studies have similarly reported that tactile stimulation administered during the second postnatal week produces more robust and long-lasting behavioral effects than stimulation delivered during other developmental periods [19]. This period coincides with substantial maturational changes in sensory processing, active exploration, and sensorimotor integration [27]. Therefore, the present findings are consistent with the possibility that the developmental timing of tactile stimulation influences subsequent sensorimotor performance.

The present results are especially relevant in view of the WAG/Rij model. Previous studies have reported that neonatal tactile stimulation reduces susceptibility to absence seizures and depression-like behavior in adult WAG/Rij rats [5] and is associated with long-lasting structural alterations in the somatosensory cortex [11]. In particular, neonatal tactile stimulation has been shown to modify dendritic spine organization in layer V pyramidal neurons of the somatosensory cortex, a region implicated in tactile processing and the pathophysiology of absence epilepsy [11]. Although the present study did not include electrophysiological, molecular, or neuroanatomical assessments, the observed improvements in distal control, gait development, and sensorimotor responses suggest that early tactile experience may influence developmental processes underlying sensorimotor function.

Another finding of the present study was a decrease in crossing time following neonatal tactile stimulation, particularly in animals stimulated during the second postnatal week. Crossing time is an integrated measure of sensorimotor performance that reflects not only locomotor ability but also the efficiency of sensory processing, motor planning, balance control, and limb coordination during task execution. Therefore, the shorter crossing times observed in the PN Week 2-NTS group are consistent with improvements in distal control, gait development, and sensorimotor responses. Accurate integration of sensorimotor information and refinement of coordinated movement patterns during the postnatal period are required for successful skilled walking tasks [13,29]. Thus, the decrease in crossing time might reflect more efficient sensorimotor processing and better movement organization.

The present study has several limitations that should be acknowledged. First, sensorimotor development was assessed at a single developmental time point (PND22). Although this time point was selected to evaluate preweaning sensorimotor maturation, longitudinal assessments across multiple developmental stages would provide a more comprehensive understanding of the temporal effects of neonatal tactile stimulation on sensorimotor development.

Second, the study focused exclusively on behavioral outcomes and did not include electrophysiological, molecular, or neuroanatomical assessments. Consequently, the neural mechanisms underlying the observed improvements in sensorimotor performance remain speculative. Furthermore, absence seizure activity was not directly measured. Therefore, it cannot be determined whether the behavioral improvements observed after neonatal tactile stimulation are associated with alterations in epileptogenesis or are independent of seizure-related mechanisms. Future studies combining behavioral assessments with electroencephalographic recordings and analyses of cortical plasticity, synaptic organization, and network activity will be necessary to clarify the biological basis of these effects.

Another limitation of the present study is the absence of a sham-handled control group. Animals receiving neonatal tactile stimulation were repeatedly removed from the home cage and subjected to handling, brief maternal separation, and exposure to a controlled external environment during the stimulation sessions. Consequently, the observed behavioral effects cannot be attributed exclusively to tactile stimulation, as they may also have been influenced by repeated handling, transient maternal separation, changes in maternal care following reunion with the dam, or other procedural factors. Previous studies have demonstrated that early-life handling and maternal separation can independently affect neurobehavioral development [1,5,11]. Moreover, in our previous study, a maternal separation/handling group that underwent neonatal handling without tactile stimulation exhibited developmental outcomes distinct from those observed after neonatal tactile stimulation, suggesting that tactile stimulation may exert effects beyond those of handling or maternal separation alone [15]. Nevertheless, because a sham-handled control group was not included in the present study, the specific contribution of tactile stimulation cannot be fully separated from the effects of repeated handling. Therefore, the findings should be interpreted as reflecting the neonatal tactile stimulation protocol as a whole rather than tactile stimulation alone. Future studies incorporating a sham-handled control group are warranted to distinguish the specific effects of tactile stimulation from those associated with handling and maternal separation.

An additional limitation is that male and female pups were analyzed together without formal assessment of sex-specific effects. Although efforts were made to balance sex distribution across the experimental groups, the study was not designed or statistically powered to evaluate potential sex differences in sensorimotor development or responsiveness to the NTS protocol. Visual inspection of the data did not suggest any obvious differences in the overall pattern of responses between male and female pups; however, these observations are descriptive only and do not support sex-specific conclusions. Future studies with larger sample sizes will be required to determine whether sex influences these developmental outcomes.

A further limitation of the present study relates to litter-related factors. Litters were not standardized to a predetermined number of pups because reducing litter size through culling may itself alter maternal care and early developmental trajectories, thereby introducing an additional experimental variable. Instead, efforts were made to ensure that dams contributing to different experimental groups had comparable litter sizes (6–8 pups), minimizing potential confounding related to litter size. Nevertheless, modest effects of natural variation in litter size on early postnatal development cannot be entirely excluded. In addition, statistical analyses were performed using individual pups as the experimental unit without explicitly accounting for litter as a random effect. Although each experimental group included offspring derived from three independent dams to minimize litter-specific maternal and environmental influences, pups from the same litter are not fully independent because they share common genetic, prenatal, and maternal environmental factors. Future studies should include a larger number of independent litters to permit mixed-effects analyses that explicitly account for litter clustering.

Finally, the study was conducted in a single genetic model of absence epilepsy. Therefore, caution is warranted when generalizing these findings to other epilepsy models or to human neurodevelopmental conditions. Despite these limitations, this study provides the first evidence of early postnatal sensorimotor alterations in WAG/Rij rat pups and demonstrates that the effects of neonatal tactile stimulation on sensorimotor maturation are strongly dependent on the developmental timing of the intervention. These findings provide a foundation for future investigations examining how early sensory experiences influence neurodevelopmental trajectories in genetic absence epilepsy.

## 5. Conclusions

In conclusion, the present study demonstrates that WAG/Rij rat pups exhibit deficits in selected aspects of sensorimotor development compared with age-matched Wistar controls as early as PND22, indicating that developmental abnormalities associated with the WAG/Rij phenotype are detectable before the classical adult expression of absence seizures. The NTS protocol was associated with improvements in distal control, gait development, sensorimotor responses, tail performance, and overall sensorimotor function. Importantly, these effects were strongly dependent on the timing of the intervention, with the second postnatal week representing a particularly sensitive developmental window for sensorimotor maturation among the developmental periods examined. Because a sham-handled control group was not included, the observed effects should be interpreted as reflecting the NTS protocol as a whole rather than tactile stimulation alone. To our knowledge, this is the first study to demonstrate both early postnatal sensorimotor deficits in WAG/Rij rat pups and developmental window-dependent effects of the NTS protocol on sensorimotor maturation in this model. These findings provide new insight into early sensorimotor development in genetic absence epilepsy and may contribute to a better understanding of the developmental alterations associated with this disorder. Future studies incorporating sham-handled control groups, longitudinal developmental assessments, complementary neurophysiological and neurobiological measures, and statistical models accounting for litter clustering are warranted to further define the specificity, underlying mechanisms, and long-term developmental effects of the NTS protocol.

## Data Availability

The data presented in this study are available within the article.

## Supplementary Materials

The following supporting information can be downloaded at: https://www.mdpi.com/article/10.3390/life16071152/s1. Table S1: Descriptive statistics (median [Q1–Q3]), Kruskal–Wallis test statistics (H), exact p-values, and epsilon-squared (ε²) effect sizes for all behavioral outcomes, including orientation, flexor/extensor activity, postural control, distal control, gait development, sensorimotor response, tail score, total sensorimotor score, and crossing time.
