# Supplementary material for: Developmental Window-Dependent Effects of Neonatal Tactile Stimulation on Sensorimotor Development in WAG/Rij Rat Pups
Source: Life (Basel). 2026 Jul 12;16(7):1152. doi: 10.3390/life16071152 (PMC13413081; doi:10.3390/life16071152)
Supplement: Supplementary file 1 [file life-16-01152-s001.zip › life-4409022-supplementary.pdf]

Table S1. Descriptive statistics, Kruskal–Wallis test results, and effect sizes for behavioral outcomes.

**Outcome**	**Wistar Control Median**	**WAG/Rij Control Median**	**PN Week 1-NTS Median**	**PN Week 2-NTS Median**	**PN Week 3-NTS Median**	**H**	**p**	**ε²**
Orientation	6 (6–6)	6 (6–6)	6 (6–6)	6 (6–6)	6 (6–6)	0.900	0.9246	0.000
Flexor/Extensor Activity	6 (6–6)	6 (6–6)	6 (6–6)	6 (6–6)	6 (6–6)	0.007	>0.9999	0.000
Postural Control	4 (4–4)	4 (4–4)	4 (4–4)	4 (4–4)	4 (4–4)	0.273	0.9914	0.000
Distal Control	6 (4.75–6.00)	4 (4–4)	4.5 (4–5)	6 (5.75–6.00)	4 (4–4)	10.770	0.0293	0.106
Gait Development	8 (8–8)	7 (7–8)	7.5 (7–8)	8 (8–8)	8 (8–8)	19.570	0.0006	0.243
Sensorimotor Response	12 (10.75–12.00)	9 (7.75–11.00)	10 (10–12)	12 (12–12)	8 (7.5–9)	37.230	<0.0001	0.519
Tail Score	2 (1–2)	1 (1–2)	2 (1.75–2.00)	2 (2–2)	2 (2–2)	10.610	0.0314	0.103
Total Sensorimotor Score	45 (43.75–46.00)	39.5 (38.75–42.00)	42.5 (40.75–44.25)	45 (43.75–46.00)	39 (38.5–42.0)	31.820	<0.0001	0.435
Crossing Time (s)	48.5 (32.0–79.0)	180 (103.25–180.0)	180 (24.25–180.0)	11 (10.0–16.25)	180 (44.0–180.0)	38.710	<0.0001	0.542

Note. Data are presented as median (Q1–Q3). H denotes the Kruskal–Wallis test statistic, p denotes the exact p-value obtained from the Kruskal–Wallis test, and ε² denotes the epsilon-squared effect size. Negative ε² values were set to 0.000, indicating negligible effect sizes.
